# Genome-Wide Identification and Expression Pattern Analysis of the DNA Methyltransferase Gene Family Revealed the DNA Methylation Response to Tapping in Rubber Tree

**DOI:** 10.3390/plants14213284

**Published:** 2025-10-27

**Authors:** Yuanyuan Hao, Bin Hu, Yongkang Xue, Xuelian Li, Kun Wang, Xuemei Dai, Zhifu Guo, Xiangyu Long

**Affiliations:** 1 State Key Laboratory of Tropical Crop Breeding, Key Laboratory of Biology and Genetic Resources of Rubber Tree, Ministry of Agriculture and Rural Affairs, Rubber Research Institute, Sanya Research Institute, Chinese Academy of Tropical Agricultural Science, Haikou 571101, China; yyhao630@163.com (Y.H.); binhu0612@foxmail.com (B.H.); x2728986359@163.com (Y.X.); wiangkuen@foxmail.com (K.W.); xuemeidai@126.com (X.D.); 2 College of Tropical Crops, Yunnan University, Puer 665099, China; 18849684649@163.com; 3 College of Biosciences and Biotechnology, Shenyang Agricultural University, Shenyang 110161, China; zfguo@syau.edu.cn

**Keywords:** *Hevea brasiliensis*, DNA methyltransferases, phylogenetic analysis, gene expression profiles

## Abstract

DNA methylation is an epigenetic modification that plays a crucial role in the regulation of gene expression, genome defense, and numerous biological processes. DNA methyltransferase (MTase) is the pivotal enzyme catalyzing the DNA methylation reaction. To explore the regulation mechanism of DNA methylation in rubber trees (*Hevea brasiliensis*), we identified 13 genome-wide MTase genes containing conserved structural domains of DNA methyltransferase based on the rubber tree reference genome. Through phylogenetic analysis, these genes were classified into four subfamilies: MET1, CMT, DRM, and DNMT2. A significant expansion of DNA methyltransferase genes was found in rubber trees, especially in the DRM subfamily. Notably, among the four members of the MET1 subfamily, only HbMET1-1 contains the complete UBA and RFD domains, suggesting its critical role in the function of MET1 in plants, while the other members may have developed different functions during evolution. Gene expression pattern analysis revealed that most DNA methyltransferases were specifically expressed at low levels in latex. However, following tapping from unharvested rubber trees, the expression levels of HbMTase genes are altered, and these alterations exhibit variability. Among them, HbMET1-1 and HbDRM2-1 exhibited a response to tapping stress, with their expression levels rapidly increasing after stress application and subsequently decreasing gradually. The expression levels of HbCMT3-1 and HbCMT3-2 continued to increase as the rubber tapping process progressed, which was consistent with the observed changes in HbMTase enzyme activity. These findings suggest that tapping, as a form of mechanical stress, affects the expression of particular genes. Through subcellular localization analysis, we found that HbDNMT2 is the only DNA methyltransferase located in the cytoplasm its expression level gradually decreases during the rubber tapping process. It is hypothesized that this gene activates a multitude of genes involved in the rubber biosynthesis pathway and participates actively in rubber biosynthesis. In conclusion, the comprehensive analysis of the structural characterization, conserved domains analysis, cis-regulatory elements, subcellular localization, expression profiling, and HbMTase enzyme activity detection provides critical insights into the functional characteristics of DNA methyltransferases in rubber trees and initially reveals the DNA methylation response in rubber tree tapping.

## 1. Introduction

Epigenetic modifications play a crucial role in regulating gene expression by altering the structure and chemical composition of DNA or chromatin, independent of sequence changes. These mechanisms include DNA methylation, histone modifications, and non-coding RNAs [1]. Among these, DNA methylation stands out as one of the earliest to be explored and is currently the most extensively studied type of modification [2,3]. This modification process is catalyzed by DNA methyltransferase, which employs S-adenosyl methionine (SAM) as the methyl (CH3-) donor to transfer CH3- to specific bases within the DNA sequence. DNA methylation is primarily classified into the following three types: methylation of the sixth carbon atom of adenine (m6A), methylation of the fifth carbon atom of cytosine (m5C), and methylation of the seventh carbon atom of guanine (m7G) [4], with m5C being the most widely studied form of DNA methylation [5].

In mammals, DNA methylation mainly occurs in the symmetrical CG context, while in plants, DNA methylation predominantly occurs primarily in three distinct sequence contexts: symmetric CG, CHG, and asymmetric CHH sites (where H = A, T, or C) [6]. There are two major patterns of DNA methylation: de novo methylation and DNA methylation maintenance. De novo methylation refers to the methylation of cytosine C-5 in the DNA strand at a new site under the action of DNA methyltransferase, independent of the existing methylated DNA strand as a template. The maintenance methylation process occurs during the semi-conservative replication of DNA and involves methylation modification at the corresponding site of the new strand [3,7]. The functional diversity of DNA methylation covers stress adaptation, developmental regulation, and secondary metabolism [8,9,10]. For instance, dynamic methylation patterns modulate responses to abiotic stressors, including cold stress [11,12], salt stress [13,14], drought stress [15,16], and heat stress [17].

Central to these processes are cytosine-5 DNA methyltransferases (C5-MTases), classified into four subfamilies: methyltransferase1 (MET1), chromatin methyltransferases (CMT), domains rearranged methyltransferase (DRM), and DNA methyltransferase 2 (DNMT2) [18]. MET and CMT are maintenance DNA methyltransferases, with MET1 primarily responsible for maintaining methylation in the CG context [19,20]. In contrast, the plant-specific CMT predominantly maintains the methylation of GHG and CHH contexts [21]. The DRM gene family is mainly involved in de novo methylation of the CG, CHG, and CHH contexts [22]. DNMT2 is involved in tRNA methylation modification and histone deacetylation [23,24,25].

Given that DNA methylation is essential for many biological processes in plants, C5-MTsae genes have been identified and characterized in several plant species. Such as *Arabidopsis thaliana* [26], castor bean (*Ricinus communis* L.) [27,28], tomato (*Solanum lycopersicum* L.) [29], kiwifruit (*Actinidia chinensis*) [30], wheat (*Triticum aestivum* L.) [31], cassava (*Manihot esculenta* Crantz) [32], and rice (*Oryza sativa* L.) [33]. Although these enzymes have been recognized in various plants, significant differences exist in both the number and function of these genes among different species. The identification and analysis of MTase genes at the whole-genome scale have been specifically undertaken for rubber trees.

Natural rubber, a strategic industrial resource, is predominantly harvested from *Hevea brasiliensis* due to its advantages, such as large rubber production, long rubber production time, and easy collection of natural rubber. Despite advances in understanding rubber biosynthesis, the epigenetic regulation of this process remains enigmatic, particularly through DNA methylation. Recent studies highlight correlations between methylation dynamics and secondary metabolite production in plants [25,34], but the correlation between rubber biosynthesis and DNA methylation modification has not been studied, and the systematic analysis of HbMTases has not been carried out.

In this study, based on the complete sequence of the rubber tree genome, we present the first genome-wide identification and characterization of C5-MTase genes in *Hevea brasiliensis*. Through integrative bioinformatics approaches, we analyze their phylogenetic relationships, conserved domains, promoter cis-elements, and expression profiles. Our research not only provides valuable information for the functional study of DNA methyltransferase genes in rubber trees but also lays a theoretical foundation for exploring the mechanisms of DNA methylation modification in rubber trees.

## 2. Results

### 2.1. Identification of HbMTaes Gene Family Members and Physicochemical Characterization Analysis

Homology-based identification of DNA methyltransferase (MTase) genes in *Hevea brasiliensis* was performed using BLASTP analysis against the *Arabidopsis thaliana* MTase protein sequences. Subsequent domain validation through the Pfam database (PF00145; DNA methylase domain) confirmed 13 HbMTase genes retaining conserved catalytic motifs, with incomplete or divergent domains excluded.

**Figure 1:**
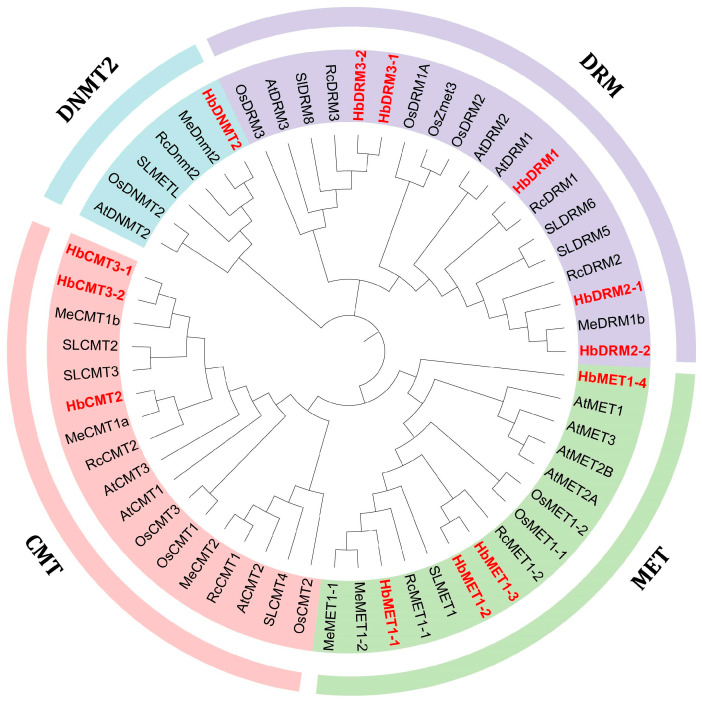
Phylogenetic analysis of MTase gene family proteins from *Arabidopsis thaliana*, tomato, cassava, castor, rice, and rubber tree. The red marks represent the genes in rubber trees.

To resolve evolutionary relationships, a maximum likelihood phylogenetic tree was constructed using MTase protein sequences from six angiosperms: *Solanum lycopersicum*, *Manihot esculenta*, *Ricinus communis*, *Oryza sativa*, *Arabidopsis thaliana*, and *Hevea brasiliensis*. The specific MTase genes information was provided in Table S3. The results revealed that the HbMTase genes were clearly divided into four distinct clades: CMT (3 members), MET1 (4 members), DRM (5 members), and DNMT2 (1 member) (Figure 1). This taxonomic framework is consistent with that of model plants such as *Arabidopsis thaliana*, suggesting strong evolutionary conservation of functional specialization.

The comprehensive analysis of 13 HbMTase protein structure revealed substantial variation in physicochemical properties (Table 1). Protein lengths ranged from 305 to 1564 amino acids (aa), with molecular weights (Mw) spanning 33.76—176.18 kDa. The largest member, HbMET1-1 (1564 aa; 176.18 kDa), contrasts with the smallest, HbMET1-3 (305 aa; 33.76 kDa). Isoelectric points (pI) exhibited a broad distribution (4.82–9.12), suggesting functional diversification in pH-dependent interactions. Hydrophobicity analysis showed average grand hydrophilic index (GRAVY) values between −0.615 and −0.185, confirming their hydrophilic nature. Stability profiling classified nine proteins as unstable (instability index >40) and four as stable.

Secondary structure prediction via SOPMA demonstrated conserved architectural patterns: random coils dominated (49.38–63.39%), followed by α-helices (25.85–43.93%) and β-sheets (6.18–11.52%). Subcellular localization predictions revealed compartment-specific distributions: HbMET, HbDRM and HbCMT subfamily members localized predominantly to the nucleus, consistent with their roles in DNA methylation maintenance, while HbDNMT2 was targeted cytoplasm (Table 2). This subcellular localization pattern is highly consistent with the MTases family members in the model plant *Arabidopsis thaliana*, indicating that the MTases gene in rubber trees has a high degree of conservation during evolution.

### 2.2. Gene Structure, Conserved Domains, and Motif Analysis of HbMTase Genes

**Figure 2:**
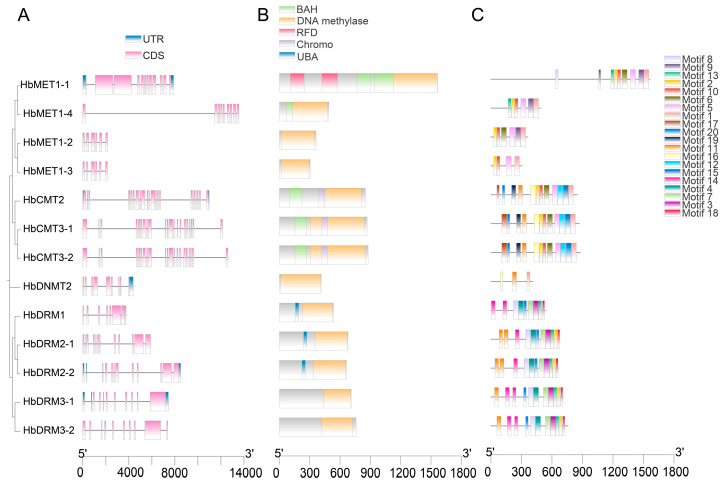
The gene structure, conserved domains, and motif distribution of HbMTase genes. (**A**): The gene structure of HbMTase genes; (**B**): The conserved domain of HbMTase genes; (**C**): The motif distribution of HbMTase genes.

The analysis of gene structure revealed significant structural diversity among members of the HbMTase family, with exon counts varying from 6 to 21. Notably, the HbCMT subfamily displayed the most intricate architecture, comprising a total of 21 exons (Figure 2A).

The conserved domain prediction results showed that all HbMTase proteins shared a conserved C-terminal DNA methylase domain responsible for SAM-dependent 5mC transfer. Subfamily-specific N-terminal domains dictated functional divergence: HbMET1-1 uniquely encoded two RFD domains (RING-finger domain). HbMET1-1, HbMET1-4, and HbCMTs harbored BAH (bromo-adjacent homology) domains. HbDRM1, HbDMR2-1, and HbDRM2-2 contained UBA (ubiquitin-associated) domains, implicating ubiquitination-dependent complex assembly in de novo methylation, suggesting that they may participate in the dynamic assembly of methylation complexes through ubiquitination signaling pathways (Figure 2B).

Motif profiling revealed subfamily-specific conservation patterns, with identical motif architectures observed within each clade, indicating that the gene domains of each subtype were highly conserved and similar in evolution, and similar gene functions may also occur during the differentiation process. Motif 17 and motif 20 unique to the CMT subfamily may correspond to BAH domains (Figure 2C, Table S2).

**Figure 3:**
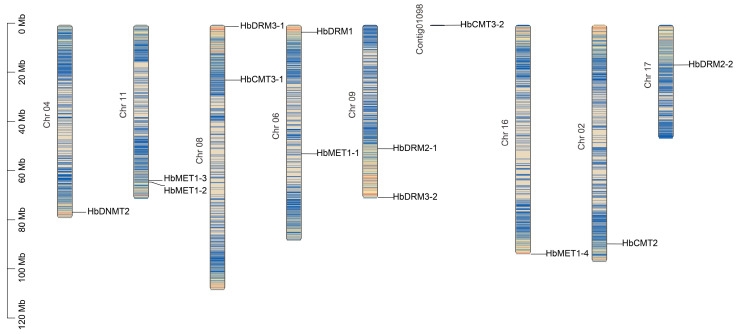
Chromosome localization of the HbMTase genes.

Chromosome localization results of the HbMTases gene family members showed that Hb-MTases were widely distributed in nine chromosomes, two genes HbMET1-2 and HbMET1-3 were located in adjacent positions on chromosome 11, which may be tandem repeats, and other gene family members were distributed in different positions of chromosomes (Figure 3).

### 2.3. Promoter Cis-Acting Elements Analysis of HbMTase Genes

**Figure 4:**
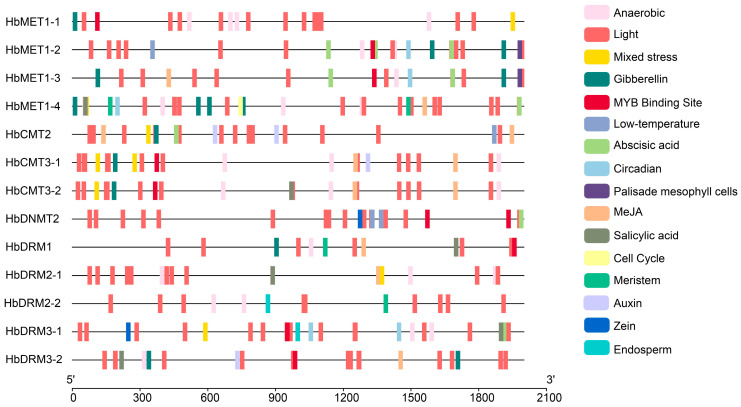
Distribution of cis-acting elements in the promoters of HbMTase genes.

**Figure 5:**
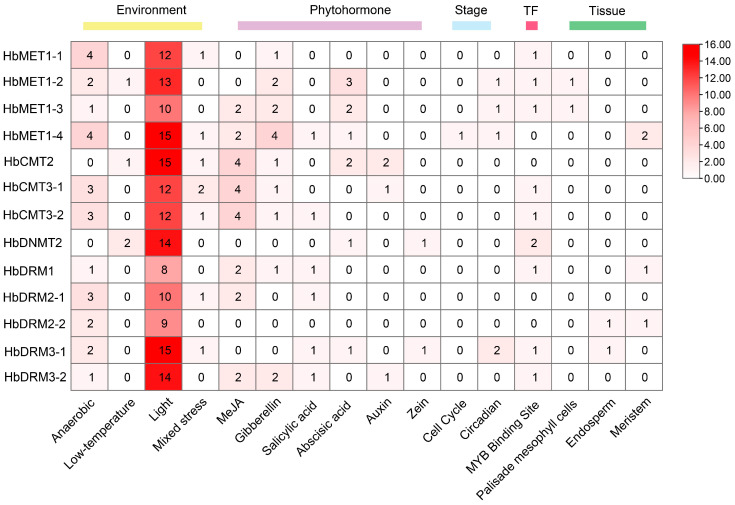
HbMTase genes promoter cis-element quantity statistics.

The prediction of expression levels can dependent on cis-regulatory elements upstream of the transcription start site (TSS). To elucidate the transcriptional regulation of HbMTase genes, we analyzed 2000 bp promoter regions upstream of the TSS across 13 HbMTase members using PlantCARE. Sixteen cis-acting elements were classified into five functional modules: environmental stress response, phytohormone signaling, developmental stage regulation, transcription factor binding (TF), and tissue-specific expression, among which environmental response elements accounted for the highest proportion (70.35%), followed by phytohormone stimulation. Notably, light-responsive elements constituted 80.71% of environmental modules, followed by anaerobic induction elements (13.20%), suggesting light-dark cycles and hypoxia as key environmental triggers. Phytohormone-related elements were dominated by methyl jasmonate (MeJA; 37.29%) and gibberellin (GA; 25.42%) (Figure 4 and Figure 5, Table S4).

### 2.4. Analysis of the Expression Patterns of the HbMTaes Genes and Determination of DNA Methyltransferase Activity

**Figure 6:**
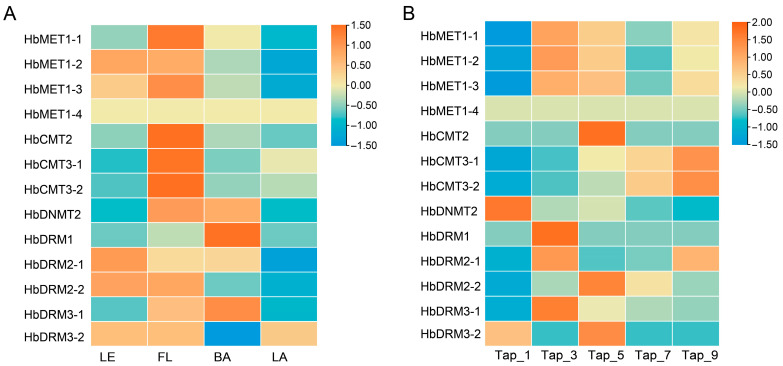
Heat map of the expression pattern of HbMTase genes. (**A**): Gene expression profiles in different tissues. LE: leaves, FL: flowers, BA: bark, LA: latex; (**B**): Gene expression profile during the rubber tapping process. Tap-1 to tap-9 represents the period from the first rubber tapping to the ninth rubber tapping. The same below.

DNA methyltransferases (MTases), as central regulators of DNA methylation, critically influence plant growth and developmental processes. To elucidate the dynamics of methylation in *Hevea brasiliensis*, we profiled the expression of 13 HbMTase genes across four tissues (mature leaves, bark, latex, and flowers) and during consecutive tappings (1st–9th) (Figure 6).

The expression characteristics of HbMTases are different in different tissues. Overall, 13 HbMTase genes were highly expressed in flowers but specifically lowly expressed in latex. Specifically, HbMET subfamily members exhibited preferential expression in leaves and flowers, with the lowest expression level in latex. HbCMT subfamily members were highly expressed in flowers but had lower expression levels in other tissues. The expression patterns of HbDRM subfamily members were different. HbDRM3-2 is specifically lowly expressed in bark, while the expression levels of HbDRM2-1, HbDRM2-2, and HbDRM3-1 are relatively lowly expressed in latex.

Distinct expression dynamics were observed among HbMTase subfamilies during the rubber tapping process, suggesting different DNA methylation regulatory mechanisms. The expression level of HbMET subfamily genes significantly increased in the early stage of rubber tapping, then gradually decreased and tended to stabilize, but the expression level was still higher than that in the early stage of rubber tapping. Members of the HbDRM family were subjected to rubber tapping stress, and their expression levels significantly increased, then gradually decreased to the expression level at the early stage of rubber tapping. Meanwhile, the expression levels of HbCMT3-1 and HbCMT3-2 genes increased directly and proportionally with the tapping process. However, the expression level of the HbDNMT2 gene continued to decline throughout the rubber tapping process. Based on the expression patterns and expression levels, we speculate that the HbMET1-1, HbCMT3-1, HbCMT3-2, HbDRM2-2 and HDNMT2 genes play significant roles in rubber biosynthesis.

**Figure 7:**
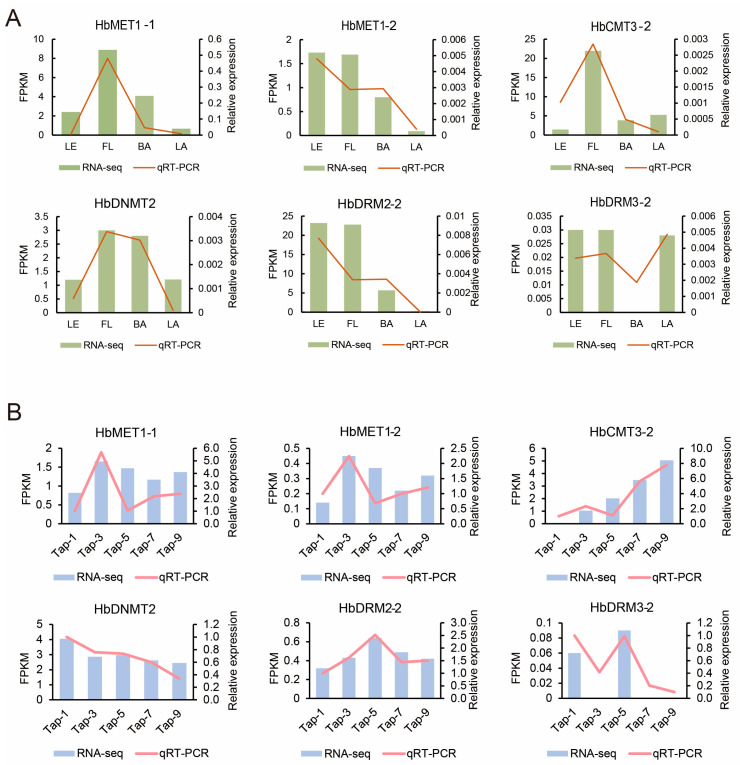
Comparative analysis of expression results between RNA-Seq and RT-qPCR for six HbMTase genes. (**A**): Comparative analysis in different tissues. (**B**): Comparative analysis during the rubber tapping process.

We selected six representative genes (HbMET1-1, HbMET1-2, HbCMT3-2, HbDRM2-2, HbDRM3-2, and HbDNMT2) for for quantitative reverse transcription polymerase chain reaction (RT-qPCR) analysis to validate the accuracy of the RNA sequencing (RNA-seq) results. The results indicated that the relative expression levels obtained from RT-qPCR were generally consistent with the fragments per kilobase of transcript per million mapped reads (FPKM) values derived from RNA-seq measurements (Figure 7), thus demonstrating the reproducibility and reliability of the RNA-seq data.

**Figure 8:**
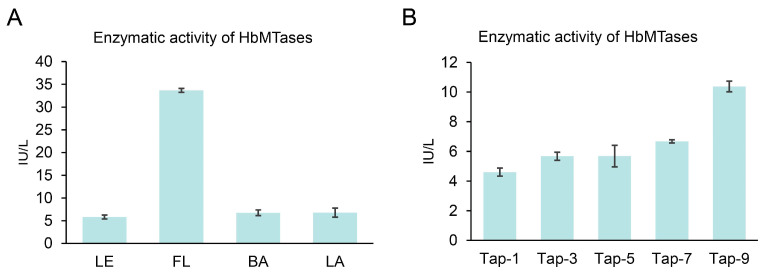
DNA methyltransferase activity in different samples of rubber trees. (**A**): Enzyme activity in different tissues. (**B**): Enzyme activity in the latex during the rubber tapping process.

In addition, we also detected the DNA methyltransferase activities in different tissues of rubber trees and during the dynamic process of rubber tapping. The activity unit of enzymes is IU/L. IU (international unit) is defined as the amount of enzyme converted per minute from 1 μmol of substrate under standard conditions (25 °C, optimal pH and substrate saturation). Therefore, IU/L represents the number of enzyme activity units contained in each liter of solution. Quantitative analysis revealed tissue-specific variation, with flower organs exhibiting significantly higher methyltransferase activity compared to other examined tissues, while no statistically discernible differences were observed among leaf, bark, and latex samples (Figure 8A). The enzyme activity gradually increased throughout the rubber tapping process and reached its peak at the ninth tapping. The activity pattern of this enzyme was positively correlated with the transcriptional profile of the key methyltransferase gene (HbCMT3-1 and HbCMT3-2), indicating that there is a synergistic regulation at the transcriptional and post-translational levels during latex biosynthesis.

### 2.5. Subcellular Localization of HbMET1-1, HbDRM2-2 and HbDNMT2

**Figure 9:**
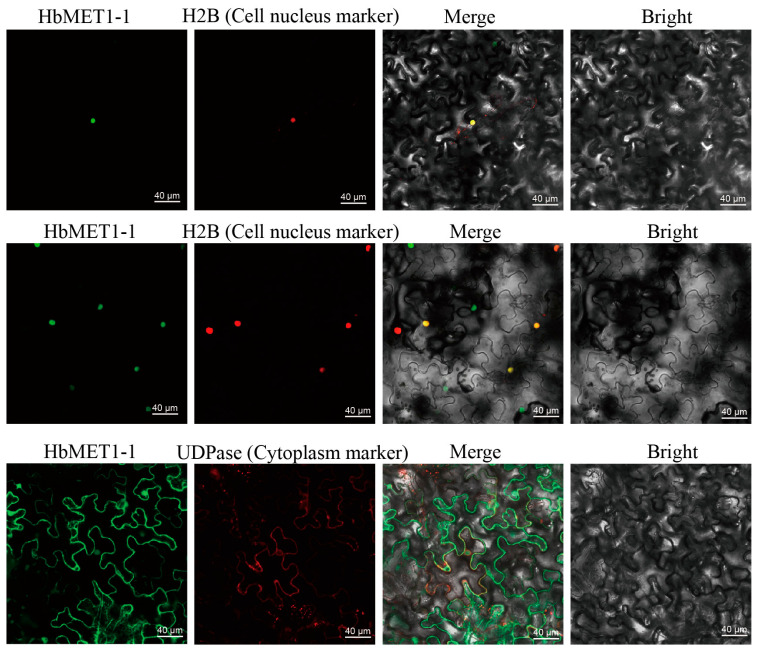
Subcellular location of HbMET1-1, HbDRM2-2 and HbDNMT2. EYFP indicates green fluorescence photography; mCherry indicates red fluorescence photography. The scale bar is 40 μm.

To determine the subcellular localization of HbMTase, HbMET1-1, HbDRM2-2 and HbDNMT2 were selected for subcellular localization analysis as they are representative members (Figure 9). The results indicated the presence of nuclear localization signals in HbMET1-1 and HbDRM2-2, suggesting their potential role in regulating gene expression by binding to specific DNA sequences in the promoter regions of target genes. Additionally, the HbDNMT2 protein was found in the cytoplasm, aligning with the predicted outcomes. These localization results support our preliminary speculation on the function of HbMTase and provide crucial functional localization information for subsequent research.

## 3. Discussion

At present, the study of DNA methyltransferase in rubber trees is relatively limited [35]. In this study, 13 HbMTase genes were identified based on the reference genome of rubber tree. Phylogenetic analysis indicated that HbMTase genes are classified into four major categories: MET, CMT, DRM and DNMT2. This classification result is highly consistent with the MTase classification framework in *Arabidopsis thaliana*, indicating that the functional differentiation of plant C5-MTase is highly conserved during the evolutionary process. Notably, the number of HbMTase genes in rubber trees was significantly higher than those of the closely related species cassava (7) [10] and castor bean (8) [28], which suggests that this gene family may have undergone a specific expansion. This expansion phenomenon may be closely associated with the high degree of specialization observed in laticifer cells, as well as the intricate regulatory requirements inherent to the rubber biosynthesis process. Specifically, the expansion of the HbMTase gene family mainly occurs in the DRM and MET1 subfamilies, and there are two copies of both HbDRM2 and HbDRM3 in rubber tree. The pairs HbDRM2-1, HbDRM2-2 and HbDRM3-1, HbDRM3-2 exhibit identical functional domains and a significant level of sequence similarity; they are well separated from one another within the genome but reside within a syntenic segment. This suggests that they likely resulted from a whole-genome duplication event. The MET1 subfamily comprises four members, among which MET1-2 and MET1-3 are located near chromosome 11. Both genes display six exons and share similar nucleotide sequences, indicating a localized duplication event may have occurred.

Although MTase genes exhibit significant variability across different species, the conserved domains present in these proteins show remarkable similarity. In this study, all members of HbMTase contain a DNA methylase structural domain, which is essential for the C5-mTase protein to accurately catalyze the methylation of cytosine at the C-5 position [3]. Furthermore, different subfamily genes also have specific N-terminal functional domains: the HbMET1-1 gene contains two BAH domains and two RFD domains, which are responsible for the recognition of hemimethylated CG dinucleotides and methylated unmodified cytosine, consistent with its critical role in the maintenance of CG methylation [36,37]. Other HbMET1 members lack these crucial N-terminal domains, suggesting that their functions may have evolved over time. All HbCMT subfamily genes include a BAH structural domain, this domain plays a vital role in gene silencing and replication by mediating interactions between chromatin and heterochromatin regions as well as facilitating protein-protein interactions [38,39]. Additionally, there exists another domain within HbCMT known as the chromo shadow domain, which is involved in chromatin interactions and is believed to mediate recognition and binding to target DNA [40,41]. DRM is a key de novo methyltransferase in plants, responsible for establishing all cytosine methylation and maintaining CHH methylation [3,42]. In this study, the HbDRM1, HbDRM2-1 and HbDRM2-2 genes in the DRM subfamily all contain the UBA structural domain, and related studies in *Arabidopsis thaliana* point out that both the DRM2 N-terminal UBA structural domain and the C-terminal methyltransferase structural domain are required for normal RNA-directed DNA methylation, supporting the essential targeting function of the UBA structural domain [43]. The HbDNMT2 gene lacks a conserved N-terminal regulatory domain and only contains a catalytic C-terminal domain. This characteristic aligns with DNMT2 proteins from other species; however, its specific function requires further investigation [44].

Cis-acting elements act as molecular switches that play a crucial role in the transcriptional regulation of stress-induced gene expression and regulate various biological processes [45]. The promoter region of the HbMTase genes were enrich in a variety of cis-acting elements, including light-responsive elements, phytohormone-responsive elements (such as methyl jasmonate, salicylic acid and abscisic acid), and stress-responsive elements. The distribution of these elements indicates that the HbMTase genes may play an important role in plant growth and development as well as stress response. For instance, the presence of light-responsive elements implies that exposure to light not only affects photosynthesis but may also influence DNA methylation levels by regulating the expression of the MTase genes, similar findings have been reported in previous studies [46,47,48]. Research has demonstrated that phytohormones can enhance the accumulation of secondary metabolites in plants. Specifically, salicylic acid (SA) and methyl jasmonate (MeJA) serve as signaling molecules involved in regulating plant immune responses and secondary metabolite production—processes often closely associated with DNA methylation [49]. Phytohormones also play a regulatory role in latex production and flow within rubber trees [50,51,52]. Furthermore, investigations have revealed that members of the CMT subfamily possess a higher number of MeJA functional elements; MeJA is critical for laticifer differentiation and rubber biosynthesis, underscoring the significance of this subfamily.

Subcellular localization analysis indicated that, except for HbDNMT2 which is located in the cytoplasm, the rest of the HbMTase genes are all located in the nucleus. This result is consistent with the characteristic that DNA methyltransferase mainly performs functions in the cell nucleus [53]. This subcellular localization varied among species, with the CsDRM2B gene localized in the cytoplasm and all other genes localized in the nucleus in the tea tree [54]. In castor, all of the DNA methyltransferases are localized in the nucleus, in addition, DRM3 is localized in chloroplasts and mitochondria, and DRM1 is also localized in mitochondria [27]. DNMT2 was the only cytoplasmic DNA methyltransferase, which was consistent with the result that the DNMT2 subcellular was located in the cytoplasm in this study [53]. Notably, DNMT2 is not only involved in DNA methylation, but has also been found to function as a tRNA methyltransferase and play an important role under stress conditions [55,56]. These results indicate that there are significant differences in the subcellular localization of DNA methyltransferases among different plant species, which may be related to their specific biological functions and adaptive evolution.

DNA methyltransferases are crucial for the establishment and maintenance of DNA methylation [57]. It is well known that DNA methylation profiles vary during the growth and development of plants and their stress response. The expression patterns of MTases also have certain differences [37,44]. In this study, we elucidated the functional differentiation of Hb-MTase genes across various tissues and during the rubber tapping process by analyzing their expression patterns in rubber trees. The transcript abundance of HbMTases demonstrated tissue-specific characteristics in different parts of the rubber tree. Notably, the HbMET gene was highly expressed in flowers, mirroring the expression patterns observed for MET members in *Arabidopsis thaliana* and rice [33,58]. Other subfamily members also exhibited high expression levels in flowers, which aligns with the elevated activity of DNA methyltransferases within floral tissues. Furthermore, both HbCMT3-1 and HbCMT3-2 showed significant expression levels in latex, suggesting they may play a vital role in rubber biosynthesis. The HbMTase genes of the same subfamily displayed similar expression profiles, indicating potential functional redundancy among these genes.

In production, the latex yield of untapped rubber trees gradually increased after tapping and stabilized after the seventh tapping. This enhancement was primarily achieved by inducing a high-abundance expression of core genes involved in rubber biosynthesis [51]. Tapping, as a mechanical stress, can be rapidly transmitted to the cell nucleus, altering the chromatin structure and thereby influencing gene expression [59]. Further expression profile analysis revealed the transcriptional pattern of the HbMTase genes during the rubber tapping process. The expression patterns of HbMET1-2 and HbMET1-3 were similar to those of HbMET1-1, indicating functional redundancy among these genes. Notably, due to its elevated expression levels and specific BAH and RFD domains, HbMET1-1 is suggested to be a key gene within the MET1 subfamily.

The members of DRM subfamily are involved in de novo cytosine methylation, and the members of this subfamily are specifically low expressed in latex. During the tapping process, the members of this gene family are induced by tapping stress, leading to significantly increased expression levels that eventually stabilize. This phenomenon indicates that the expansion of the DRM subfamily may be closely related to the rubber biosynthesis process, regulating the DNA methylation level during the rubber tapping process through de novo methylation patterns in response to tapping stress. Notably, the expression pattern of HbDMR3-2 gene in the DMR subfamily is different from that of other genes, and it is speculated that different regulation patterns may also exist in the DMR subfamily genes, which is consistent with the results of other species [30]. Furthermore, both HbDMR3-1 and HbDMR3-2 lack characteristic UBA domains found in other members of their respective DMR subfamily; this absence may further elucidate differences in their expression patterns compared to other DRM members. In the CMT subfamily, the expression levels of HbCMT3-2 and HbCMT3-1 genes exhibited a gradual increase throughout the tapping process, whereas the expression level of HbCMT2 remained relatively low. In *Arabidopsis thaliana*, CMT2 and CMT3 are known to differ in their preferred target sequences; specifically, CMT2 primarily maintains the methylation level of CHH sequences [43]. The aforementioned results suggest that CHH plays a crucial role in sustaining methylation during rubber biosynthesis. Conversely, the expression level of the HbDNMT2 gene gradually decreased during the rubber tapping process. Notably, HbDNMT2 is identified as the only DNA methyltransferase located within the cytoplasm of rubber trees. Natural rubber biosynthesis occurs in the cytoplasm of laticiferous cells. Furthermore, latex production from unharvested rubber trees progressively increased following tapping, indicating that this practice promotes rubber biosynthesis. Therefore, we speculate that rubber tapping leads to a decrease in the expression level of the HbDNMT2 gene, which subsequently activates the expression of key genes involved in the rubber biosynthesis pathway and enhances rubber yield. Furthermore, during the rubber tapping process, the enzymatic activity of DNA methyltransferase progressively increases with an increasing number of tapping events. This pattern is contrary to that observed for the HbDNMT2 gene but aligns with the expression patterns of both HbCMT3-2 and HbCMT3-1 genes. We propose that rubber tapping acts as a stress response mechanism that elevates DNA methyltransferase activity following stress induction. This process inhibits the expression of certain genes, closely associated with HbCMT3-2 and HbCMT3-1 genes. Conversely, within the cytoplasm, rubber tapping facilitates rubber biosynthesis, potentially regulated by the HbDNMT2 gene. It should be noted that DNA methylation is not tissue-specific but cell type-specific traits, different cell types have different gene expression levels and may have different methylation activities. In the subsequent research, we will attempt to solve this problem through direct in situ methylation analysis. In conclusion, the expression pattern of MTases genes in rubber trees not only reflects their functional differentiation across various tissues but also highlights their potential roles in both rubber biosynthesis and tapping processes.

## 4. Materials and Methods

### 4.1. Identification and Phylogenetic Analysis of the MTase Genes

To identify putative DNA methyltransferase (MTase) genes in the rubber tree (*Hevea brasiliensis*), we employed a dual strategy combining homology-based searches and domain validation. First, protein sequences of the MTase family from *Arabidopsis thaliana* were used as the reference in a BLASTP (v2.16.0) analysis against the *Hevea brasiliensis* genome (NGDC, https://ngdc.cncb.ac.cn/, PRJCA021235, accessed on 13 December 2024). Sequences with significant homology (E-value < 1 × 10^−20^, coverage > 50%) were retained as candidate HbMTases. Second, the Hidden Markov Model (HMM) profile of the DNA methyltransferase domain (PF00145) was downloaded from the Pfam database (http://pfam.xfam.org/, accessed on 13 December 2024) and applied to screen the *Hevea brasiliensis* proteome using HMMER v3.3.2. Genes identified through both approaches were considered as members of the DNA methyltransferase family in *Hevea brasiliensis* (HbMTases).

To resolve evolutionary relationships among DNA methyltransferases, a phylogenetic tree was reconstructed using the Neighbor-Joining (NJ) algorithm in MEGA-X (v11.0.5). Branch support was assessed through bootstrap resampling with 1000 replicates, and nodes with bootstrap values ≥ 70% were considered strongly supported.

### 4.2. Physicochemical Characterization Analysis and Protein Secondary Structure Prediction

The ExPASy online tool (http://expasy.org/, accessed on 13 January 2025) was used to predict and analyze the molecular weight, isoelectric point, and other physicochemical characterization of the gene family members. The subcellular localization of Hb-MTase proteins were performed via Plant-mPLoc (http://www.csbio.sjtu.edu.cn/bioinf/plant-multi/, accessed on 13 January 2025). Secondary structure composition (α-helices, β-sheets, and random coils) was analyzed using the SOPMA algorithm (https://npsa.lyon.inserm.fr/cgi-bin/npsa_automat.pl?page=/NPSA/npsa_sopma.html, accessed on 13 January 2025).

### 4.3. Gene Structure Analysis, Conserved Domain Prediction, Motif Prediction, and Chromosome Localization

Gene structures of HbMTase family members were analyzed by aligning coding sequences (CDS) with genomic DNA using the Gene Feature Format version 3 (GFF3) annotation file. Chromosomal localization was mapped to the *Hevea brasiliensis* genome assembly via TBtools (v2.030) [60].

Conserved protein domains were predicted through the NCBI Conserved Domain Database (CDD; https://www.ncbi.nlm.nih.gov/cdd/, accessed on 15 January 2025) with an E-value cutoff of 1 × 10^−5^. Motif analysis was conducted utilizing the MEME suite v5.5.7 (http://meme-suite.org/, accessed on 15 January 2025) under the following parameters: motif width range = 6–50 amino acids, maximum motifs = 20, and site distribution = zero or one occurrence per sequence (zoops). The resulting motifs were annotated and visualized via TBtools (v2.030).

### 4.4. Analysis of the Promoter Cis-Regulating Elements

The 2000 bp DNA sequence upstream of the transcriptional start sites (TSS) of HbMTase genes was extracted from the *Hevea brasiliensis* genome using TBtools (v2.030). Cis-regulatory elements were predicted via PlantCARE (http://bioinformatics.psb.ugent.be/webtools/plantcare/html/, accessed on 15 January 2025) with default parameters. Results were visualized through TBtools (v2.030), using hierarchical clustering based on element type frequency, with color-coded heatmaps generated to highlight regulatory patterns.

### 4.5. RNA Sequencing

The rubber tree cultivar ‘Reyan 73397’ (Danzhou, China) was used for transcriptome analysis. The samples were derived from different tissues (flowers, mature leaves, latex, and bark) of 10-year-old rubber trees, as well as latex from unharvested rubber trees after continuous tapping (1st, 3rd, 5th, 7th, and 9th tapping). For each time point, three independent biological replicates were harvested. All samples were immediately frozen in liquid nitrogen and stored at −80 °C until RNA extraction. RNA-seq libraries were prepared and sequenced by Shanghai Meiji Biomedical Technology Co., Ltd. (Shanghai, China). The FPKM (fragment per kilobase of transcript per million mapped reads) value was calculated for gene expression levels.

### 4.6. Gene Expression Profiles and Enzymatic Activity Assays

Total RNA was extracted from various tissues of *Hevea brasiliensis* (mature leaves, bark, latex, and flowers), and latex samples were collected during sequential tapping (1st–9th tapping) of the ‘Reyan 7-33-97’ cultivar using a plant RNA extraction kit. cDNA synthesis was performed using the PrimeScript™ RT Reagent Kit (Takara Bio, Dalian, China) according to manufacturer guidelines.

Quantitative reverse transcription PCR (RT-qPCR) analysis was performed utilizing YLS 8 as an internal control alongside gene-specific primers designed via Primer-BLAST (NCBI). Relative expression levels were calculated employing the 2^−ΔΔCT^ method. The detailed primer information is provided in Table S1. The enzymatic activity of DNA methyltransferase (DNMT) was quantified using the Plant DNMT ELISA Kit (Colorimetric; Shanghai Fusheng Industrial Co., Ltd., Shanghai, China, A126664), following the manufacturer’s protocol. Three independent biological replicates were utilized for this experiment.

### 4.7. Subcellular Localization

The full-length CDS of HbMET1-1, HbDRM2-2, and HbDNMT2 was cloned and ligated into the multiple cloning sites of the pEarley-EYFP vector, resulting in the formation of the recombinant pEarley-HbMET1-1-EYFP, pEarley-HbDRM2-2-EYFP, and pEarley-HbDNMT2-EYFP plasmids. Subsequently, equal volumes of pEarley-HbMET1-1-EYFP and pEarley-HbDRM2-2-EYFP were mixed with the nucleus localization fusion protein AtH2B-mCherry. In addition, an equal volume mixture was prepared using pEarly-HbDNMT2-EYFP with the cytoplasmic localization fusion protein AtUDPase-mCherry. The resulting bacterial suspensions were then injected into the abaxial side of 4-week-old tobacco leaves. After a dark incubation period of three days, EYFP fluorescence was observed under a laser confocal microscope using 514 nm excitation light, while mCherry fluorescence was detected under 587 nm excitation light.

## 5. Conclusions

In this study, we conducted a genome-wide identification and expression pattern analysis of the methyltransferase (MTase) family members in rubber trees. The results revealed the presence of 13 HbMTase genes in rubber trees, which were classified into four subgroups based on their phylogenetic relationships: MET1, CMT, DNMT2, and DRM. The promoter regions of HbMTase genes contain a variety of cis-acting elements, including photoresponsive elements, hormone-responsive elements, and stress-responsive elements. Subcellular localization analysis indicated that all HbMTase genes were localized within the nucleus except for HbDNMT2, which was found in the cytoplasm. Additionally, we investigated the expression patterns of HbMTase genes in different tissues and during the rubber tapping process. It appears that HbDNMT2 may play an important role in rubber biosynthesis.

In conclusion, this study identified 13 HbMTase genes in rubber trees, and we conducted a comprehensive analysis of their gene structures, conserved domains, cis-acting elements, and expression patterns. The findings from this research provide a valuable foundation for further elucidating the mechanisms underlying DNA methylation processes involved in rubber biosynthesis.

## Figures and Tables

**Table 1:** Physicochemical characterization of the MTase genes in rubber tree.

Gene Name	Gene ID	Protein Length/aa	Mw/Da	pI	Instability Coefficient	GRAVY	Aliphatic Index
HbMET1-1	EVM0040483.1	1564	176,182.09	6.28	44.78	−0.5	76.14
HbMET1-2	EVM0017774.1	363	40,470.56	7.95	40.69	−0.204	78.18
HbMET1-3	EVM0034152.1	305	33,775.82	8.01	37.8	−0.185	81.25
HbMET1-4	EVM0009780.1	489	54,555.93	9.12	37.2	−0.41	81.6
HbCMT2	EVM0022511.1	851	95,824.44	5.46	39.61	−0.541	75.86
HbCMT3-1	EVM0032926.1	868	98,273.59	5.11	41.15	−0.615	74.91
HbCMT3-2	EVM0037387.1	878	99,130.47	5	41.19	−0.604	76.49
HbDRM1	EVM0009056.1	535	60,795.16	5.07	44.7	−0.411	83.29
HbDRM2-1	EVM0008822.1	679	76,276.52	5	39.63	−0.282	88.32
HbDRM2-2	EVM0008920.5	663	74,009.45	5.03	43.31	−0.406	79.29
HbDRM3-1	EVM0030405.1	710	80,273.46	4.82	48.68	−0.465	77.96
HbDRM3-2	EVM0010462.1	759	85,960.17	5.2	51.85	−0.433	78.59
HbDNMT2	EVM0039861.1	413	46,859.4	5.83	41.76	−0.198	83.78

Mw: Molecular Weight; pI: Isoelectric Point; Da: Dalton; aa: Amino Acid; GRAVY: Average hydrophilic coefficient.

**Table 2:** Subcellular localization and secondary structure prediction of MTase genes in rubber tree.

Gene Name	Gene ID	Subcellular Localization	α-Helix (%)	β-Fold (%)	Random Curling (%)
HbMET1-1	EVM0040483.1	Cell nucleus	29.03	13.11	57.86
HbMET1-2	EVM0017774.1	Cell nucleus	30.3	12.4	57.3
HbMET1-3	EVM0034152.1	Cell nucleus	33.77	11.15	55.08
HbMET1-4	EVM0009780.1	Cell nucleus	27.4	9.2	63.39
HbCMT2	EVM0022511.1	Cell nucleus	27.85	10.46	61.69
HbCMT3-1	EVM0032926.1	Cell nucleus	26.27	11.52	62.21
HbCMT3-2	EVM0037387.1	Cell nucleus	25.85	8.54	65.6
HbDRM1	EVM0009056.1	Cell nucleus	43.93	6.73	49.35
HbDRM2-1	EVM0008822.1	Cell nucleus	43.45	7.07	49.48
HbDRM2-2	EVM0008920.5	Cell nucleus	42.38	6.18	51.43
HbDRM3-1	EVM0030405.1	Cell nucleus	38.03	6.76	55.21
HbDRM3-2	EVM0010462.1	Cell nucleus	34.78	8.83	56.39
HbDNMT2	EVM0039861.1	Cytoplasm	28.33	9.69	61.99

## Data Availability

Data are contained within the article and Supplementary Materials.

## Supplementary Materials

The following supporting information can be downloaded at: https://www.mdpi.com/article/10.3390/plants14213284/s1. Table S1. Primers used for RT-qPCR analysis, Table S2. Distribution of conserved motifs in Hb-MTase based on the results of MEME (http://meme-suite.org/) analysis, Table S3. Information on MTase genes in the five tested species, Table S4. Number of cis-elements in the promoter region of HbMTase genes.
